# An Ultra-Broadband and Highly-Efficient Metamaterial Absorber with Stand-Up Gradient Impedance Graphene Films

**DOI:** 10.3390/ma16041617

**Published:** 2023-02-15

**Authors:** Bian Wu, Biao Chen, Shuai Ma, Ding Zhang, Hao-Ran Zu

**Affiliations:** National Key Laboratory of Antennas and Microwave Technology, Xidian University, Xi’an 710071, China

**Keywords:** metamaterial absorber, graphene films, ultra-broadband absorber

## Abstract

Metamaterial absorbers (MMAs) that absorb electromagnetic waves among an ultra-broad frequency band have attracted great attention in military and civilian applications. In this paper, an ultra-broadband and highly-efficient MMA is presented. The unit cell of the proposed MMA was constructed with two cross-placed stand-up gradient impedance graphene films, which play a key role in improving impedance matching. Considering the trade-off between absorbing performance and processing complexity, in our design, we adopted the stand-up graphene films that have a gradient with three orders of magnitude in total. The simulated results of the proposed absorber show an ultra-broadband absorption (absorptivity > 90%) from 1.8 GHz to 66.7 GHz and a highly-efficient absorption (absorptivity > 97%) in the range of 2–21.7 GHz and 39.6–57 GHz. The field analysis was adopted to explain the mechanism of the proposed absorber. To validate this design, a prototype of 20 × 20 units was processed and assembled. The graphene films were processed with graphene conductive ink using screen print technology. The measured results are in good agreement with the simulated ones. The proposed absorber may find potential applications in the field of stealth technologies and electromagnetic interference.

## 1. Introduction

Metamaterials are arrays of the periodic arrangement of subwavelength artificial units, with the electromagnetic properties undiscovered in natural mediums. It has attracted more and more attention because of its ability to control electromagnetic energy arbitrarily, and a large number of radio frequency devices based on metamaterials have emerged. By designing the structure of unit cells, the electric or magnetic resonance will occur in the metamaterials at the desired frequency band. The incident electromagnetic wave will enter the metamaterials without reflections and be stored in it. Since all parts of the structure are lossy, such as metal, substrate, and lumped elements, the incident energy will be absorbed and lost by absorbers. MMAs are widely used in the field of stealth technologies and electromagnetic interference/compatibility for their flexible design. Compared with traditional absorbing materials, the reported MMAs usually have a broader operating band and a lighter weight. Moreover, the center frequency could also be designed to the desired one easily. However, the absorptivity of traditional absorbing materials is higher than that of MMAs. So, it is important for MMA to achieve an ultra-broadband and highly-efficient absorption, which is an essential indicator of absorbers for practical applications.

One of the most classic microwave absorbers, the Salisbury absorption screen [1], is composed of a resistance layer on the surface, a quarter-wavelength dielectric isolation layer, and a metal backboard. The impedance of the surface resistance layer is matched with the free space at center frequency and the incident electromagnetic wave enters the absorbing structures without any reflection. Since the physical thickness of the Salisbury screen is wavelength-dependent, it just operates at several relatively narrow frequency bands around basic frequency and high order frequencies. In recent years, some researchers have designed MMAs with wideband absorption by the three-dimensional structure. In [2], an optically transparent broadband high-efficiency microwave absorber using standing-up circular split rings was proposed. The absorber realizes a 99% absorptivity in the frequency ranging from 4.6 GHz to 9.3 GHz (with the relative absorption bandwidth exceeding 68%). The absorptivity is high but the bandwidth is not broad enough. In [3], a three-dimensional lightweight metamaterial with ultra-wideband microwave absorption was proposed and it can absorb over 90% of the incident wave energy in the range of 1.46–40 GHz with the 3D cross array structure. In [4], a three-dimensional microwave metamaterial absorber based on a vertical circular lattice array realizes a 90% absorptivity in the range of 3.5–31.8 GHz and a 95% absorptivity in the range of 4–31 GHz. From the reported literature above, compared with planar MMAs, three-dimensional absorbers have a wider absorption bandwidth at the cost of increasing the profile height. Nowadays, the electromagnetic environment is becoming more and more complicated, which puts forward more stringent requirements on MMAs. Therefore, it is meaningful to improve the absorption rate and the bandwidth of the MMAs while keeping the profile.

Graphene, as one of the most promising materials in the design of MMAs, has attracted wide attention in recent years [5,6]. Since its conductivity can be easily modulated by external voltages, a number of tunable graphene-based MMAs operating in the microwave band [7,8], mid-infrared band [9], and terahertz band [10] have been emerging. However, the bandwidths of graphene-based MMAs are usually narrow. To broaden the bandwidth, most of the reported works adopted the method of stacking patterned multiple resonant structures [11,12,13], while the bandwidths of absorbers with 2D planar structures are still limited.

In this paper, an ultra-broadband and highly-efficient metamaterial absorber with stand-up gradient impedance graphene films is proposed. This paper is arranged as follows. In Section 2, the design of the proposed absorber is presented and analyzed. With impedance matching theory, the gradient impedance graphene resistive films were adopted to the design of MMA. To show the superiority of the proposed gradient impedance films, we give the simulation results of the MMAs with stand-up uniform graphene resistive films as a comparison. Then, to illustrate the mechanism of the proposed MMA, the field analysis was adopted. Form field distributions at the selected frequency, we analyzed how it can absorb electromagnetic waves efficiently during an ultra-broad band. In Section 3, a prototype of 20 × 20 units was processed using graphene conductive ink and screen print technology. The adopted process is easy, effective and low-cost, which is suitable for practical applications. The S parameter measurement was carried out in two frequency bands with two horn antennas of 2–18 GHz and 18–40 GHz, respectively. Finally, we present a summary in Section 4.

## 2. Structure Design and Analysis

### 2.1. Structure and Simulation Results

**Figure 1:**
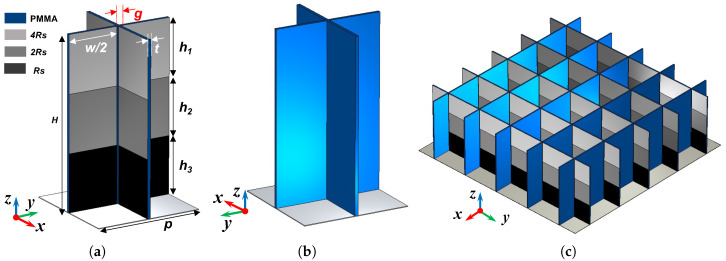
Geometry of the proposed MMA. (**a**) Perspective view of one unit cell. (**b**) Perspective view of one unit cell in the opposite direction. (**c**) Perspective view of absorber.

In this part, we will present the proposed design and its performance in detail. We will indicate how this idea emerges as well. Firstly, the structures of the proposed MMA are presented in Figure 1.

The unit cell, as shown in Figure 1a, is constructed with several graphene films with stand-up polymethyl methacrylate (PMMA) with a thickness of *t* as support. The $\epsilon_{r}$ and tanδ of PMMA in simulations were 2.7 and 0.0083, respectively. The period of the unit cell and the gap between two columns of films were denoted as *p* and *g*, respectively. The view in Figure 1b shows that the films are just attached on one single side of PMMA. Moreover, please note that the sheet resistances of graphene films on PMMA vary in a gradient along the direction of incidence. The sheet resistances of graphene films were 4$R_{s}$, 2$R_{s}$ and $R_{s}$ from top to bottom in our design. After parameter analysis, $R_{s}$ was chosen as 100 Ω/□ in this paper. The varying sheet resistance leads to different port impedances at different heights; thus, we named it ‘gradient impedance graphene film’. All parameters related to the proposed MMA are recorded in Table 1.

**Figure 2:**
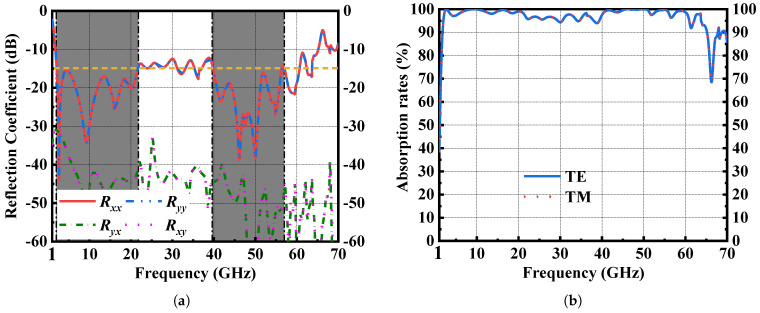
(**a**) Simulated co–polarization and cross–polarization reflection coefficients of the proposed absorber under normal incident wave with x and y polarization. (**b**) Corresponding absorption rate.

In the case of an extreme broadband frequency response, the results calculated by the Time Domain Solver are more accurate. So, the full-wave simulation was performed on CST Studio Suite 2021 with the Time Domain Solver in this paper. The simulation results under the TE and TM polarized incident wave are shown in Figure 2. For absorbers, there is almost no transmission wave due to the metallic backboard. So, the transmission coefficient $|S_{21}|$ approximates to 0 and the absorptivity is given by

$$A=1-|S_{11}{|}^{2}-|S_{21}{|}^{2}=1-{\left|S_{11}\right|}^{2}=1-|R_{co}{|}^{2}-{\left|R_{cross}\right|}^{2},$$

where $S_{11}$ is the overall reflection coefficient including co-polarization ($R_{co}$) and cross-polarization ($R_{cross}$) reflections.

As shown in Figure 2a, the magnitude of $R_{cross}$ under x and y polarizations is small enough to ignore. Therefore, when $S_{11}$ is lower than −10 dB, the absorptivity is greater than 90%. When $S_{11}$ is lower than −15 dB, it means that the absorptivity is greater than 97%. From Figure 2b, the proposed MMA shows an ultra-broadband absorption with an absorptivity larger than 90% from 1.8 GHz to 66.7 GHz. Moreover, in the range of 2–21.7 GHz and 39.6–57 GHz, a highly-efficient absorption with an absorptivity larger than 97% is realized.

**Figure 3:**
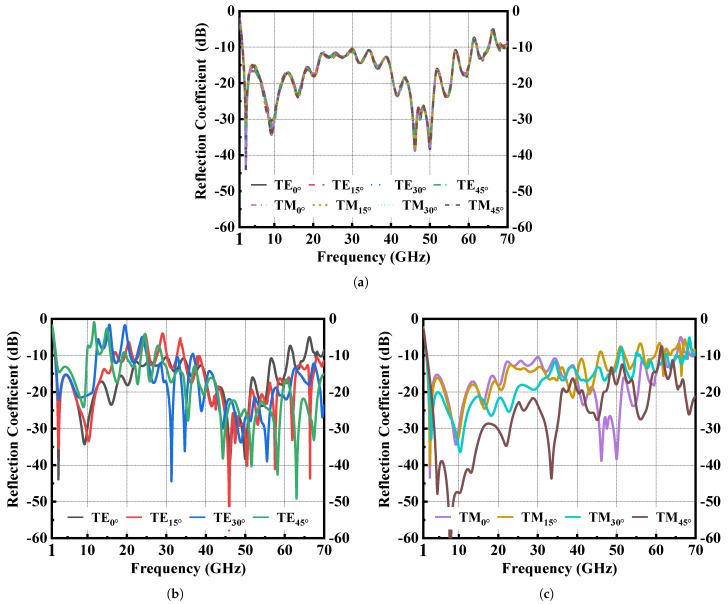
Simulated co–polarization reflection coefficient of the proposed absorber (**a**) Under normal incident wave with varying polarization. (**b**) Under oblique incident TE wave. (**c**) Under oblique incident TM wave.

Figure 3 shows a simulated performance of the proposed MMA under oblique incidence and varying polarization. Since we identified that the cross-polarization reflection of proposed MMA is small enough to ignore, we plotted the simulated co-polarization reflections in Figure 3. The results in Figure 3a show that the proposed absorber is polarization insensitive. Under the oblique incidence of TE polarization in Figure 3b, the operating bandwidth gradually decreases with the increase of incident angle. While under TM polarization in Figure 3c, the bandwidth broadens and the absorptivity becomes larger with the increase of incident angle.

### 2.2. Gradient Impedance

**Figure 4:**
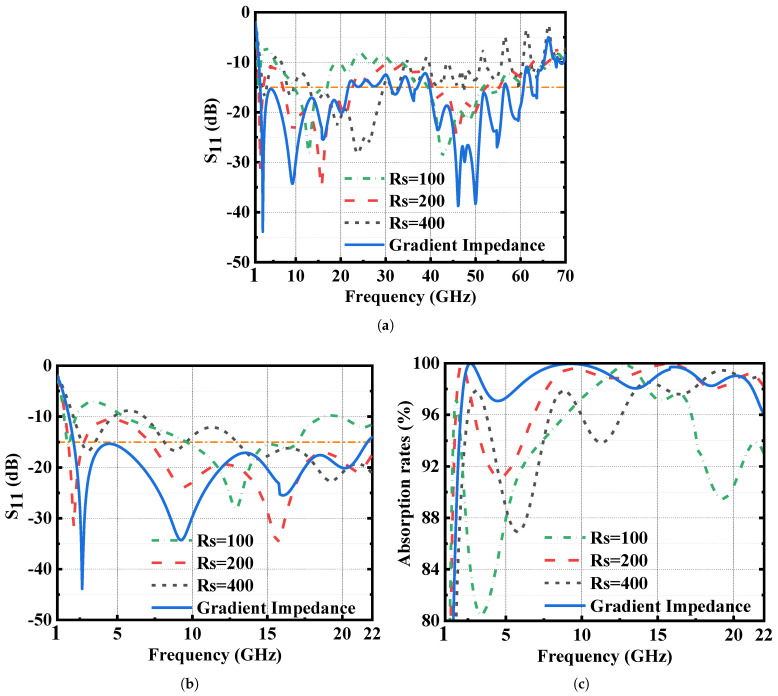
Comparison between absorbers with gradient and uniform films. (**a**) Simulated $S_{11}$ among 1–70 GHz. (**b**) Simulated $S_{11}$ among 1–22 GHz. (**c**) Simulated absorptivity among 1–22 GHz.

To demonstrate the superiority of the proposed MMA over the reported 3D absorbers with stand-up uniform resistive films [3,14], we have performed three more groups of simulations and the corresponding results are plotted in Figure 4a together. To show the improvement clearly, we present the simulated results from 2 GHz to 22 GHz in Figure 4b,c as well. Like the reported 3D absorbers, all these four MMAs exhibit a broadband absorption of over 90% absorptivity. However, in Figure 4a, we can see that it is hard to achieve a broadband and highly-efficient absorption simultaneously with stand-up uniform resistive films. This is because we cannot establish a perfect impedance matching via resistive films with just one sheet resistance among an ultra-broad band.

**Figure 5:**
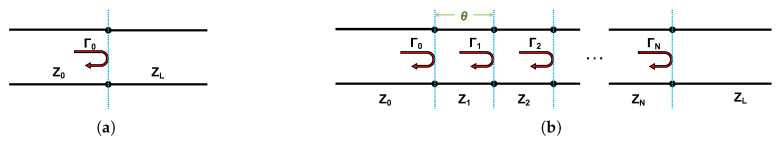
(**a**) Reflection caused by distinct characteristic impedance. (**b**) Multisection transformer from the theory of small reflections.

The theory of small reflections [15] proves that we can use multiple segments of transmission lines with gradient characteristic impedances to match two different characteristic impedances well, and the schematic is depicted in Figure 5. For an N-order Multisection transformer, the reflection coefficient is given:

$$\begin{array}{c} \Gamma \left(\theta \right)=e^{-jN\theta }\left\{\Gamma_{0}\left[e^{jN\theta }+e^{-jN\theta }\right]+\Gamma_{1}\left[e^{j(N-2)\theta }+e^{-j(N-2)\theta }\right]+\ldots \right\}\\ =2e^{-jN\theta }\left[\Gamma_{0}\cos N\theta +\Gamma_{1}\cos\left(N-2\right)\theta +\ldots +\Gamma_{N}\cos\left(N-2n\right)\theta +\ldots +\frac{1}{2}\Gamma_{N/2}\right]. \end{array}$$

The importance of the results above lies in the fact that we can synthesize any desired reflection coefficient response as a function of frequency (θ) by properly choosing the $\Gamma_{N}$ and using enough sections (*N*). If we increase *N*, the step changes in the characteristic impedance between the sections would become smaller, and the transformer geometry approaches a continuously tapered line. Please note that, since the electrical length (θ) is related to the frequency, there is a trade-off between bandwidth and in-band reflection coefficient. However, the matching between transmission lines is a little bit different in the situation of the proposed MMA. The stand-up uniform resistive film structure has the feature of broadband already, which means the matching between free space and absorbers is good. So, the reflection is originally small and the gradient impedance is adopted to improve this good matching. Under this circumstance, the ‘mismatch’ between free space and MMA is small enough. This is why we can obtain a highly-efficient absorption band without sacrificing the bandwidth.

From this idea, we used graphene films with 3-order gradient sheet resistances to construct gradient impedance, which improved the impedance matching between the free space and absorbers. We all know that the higher orders of the gradient, the better the matching that will be realized. There are two reasons we chose a 3-order to design the absorber. On the one hand, there are many parameters which impact the absorbing performance, especially the sheet resistance of each film. It is difficult to optimize so many parameters if the order is chosen to be too high. We chose the order to suit the requirements and to identify this method. On the other hand, the complexity and the cost of the process will increase as the order increases. The reason we set the sheet resistance of three graphene films to a multiplicative relationship was to facilitate the process as well, which will be explained in Section 3 in detail.

### 2.3. Mechanism of Proposed MMA

**Figure 6:**
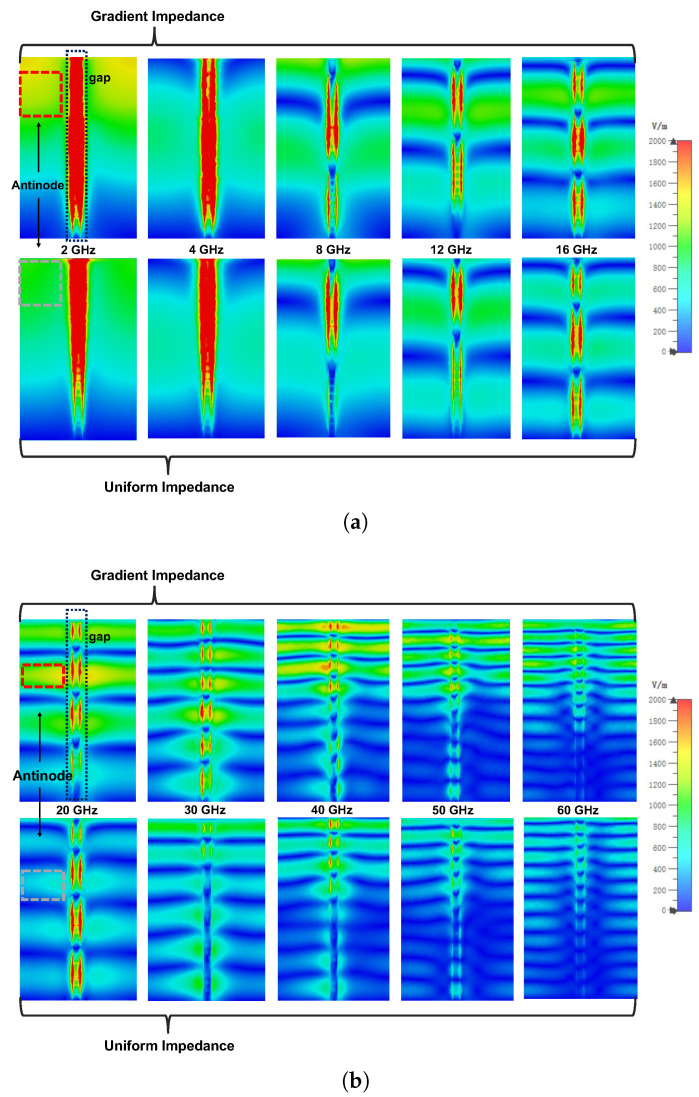
Electrical field distributions at different frequencies. Upper row is gradient impedance with 400, 200, and 100 Ω/□. Lower row is uniform impedance with 200 Ω/□. (**a**) 2–16 GHz. (**b**) 20–60 GHz.

In this part, we will illustrate the mechanism of the proposed metamaterial absorber with the method of field analysis. From the array structure introduced in Figure 1c, the stand-up films enclosed a lot of cavities with one opening. In Figure 6, the distinct antinodes and nodes reveal that there are many standing wave modes. On the one hand, the wave goes through lossy films and the energy dissipates effectively at the positions of antinodes. On the other hand, due to the narrow gap between neighboring films, the stronger electrical field at the gaps is excited and results in a large loss at the edges of graphene films. In addition, the comparison of electrical field distributions in Figure 6 suggests that a better matching is achieved by the proposed gradient impedance structure. If we focus on the amplitude of the electrical field at the positions of antinodes labelled with red and gray dashed boxes, we will find that the electrical field amplitude on the gradient impedance structure is larger than that of nthe uniform impedance ones, which means a larger loss is caused by gradient resistive films.

In summary, the gap is narrow compared to the wavelength at a low frequency. The capacitance effect caused by the gap is strong, which leads to a strong electrical field at the gap. Therefore, at a low frequency, the loss is mainly caused by two edges of graphene films around the gap. At a high frequency, compared to the wavelength, the gap is wide. The capacitance effect caused by the gap becomes weaker, which means that the loss caused by the gap decreases. However, the electrical length increases with increasing frequency. Stand-up graphene films are lossy materials and we can regard them as a lossy transmission line. A larger electrical length means a longer lossy path. The energy dissipation caused by a standing wave becomes comparable to that caused by the gap. So, the loss is caused by two factors at a high frequency. Critically, the absorptivity of our structure is improved during an ultra-broad band due to the better matching brought by the gradient impedance structure.

## 3. Fabrication, Measurement and Discussion

### 3.1. Fabrication Details

To verify the proposed absorber with gradient impedance graphene films, a prototype constructed with 20 × 20 units was fabricated and measured. In this paper, the graphene films were processed with graphene ink using screen-printing technology. The preparation and characterizations of graphene conductive ink have been reported in our previous works [16,17,18]. The raw material proportioning in this paper was modified and is given in Table 2. In the fabrication of the prototype, graphene films with three different sheet resistances are required. The sheet resistance of films ($R_{s}$) is determined by the conductivity of materials (σ) and the thickness of films (*t*). So, we can change the sheet resistances of graphene films by adjusting the film thickness.

$$R=\frac{1}{\sigma *t}.$$

In terms of screen-printing technology, the thickness of printed film is affected by the mesh density of the screen, the printing force/angle, and the viscosity of ink. However, it was difficult for us to control these parameters precisely since we printed films manually with a manual screen printer. Finally, to facilitate processing and increase precision, we controlled the sheet resistances of graphene films by the number of print passes, because increasing the times of printing results in an increase in thickness. For example, in this paper, the graphene films with sheet resistances of 400, 200, and 100 correspond to 1, 2, and 4 print passes, respectively. That is why we keep the sheet resistances of graphene as a multiplicative relationship in simulations.

**Figure 7:**
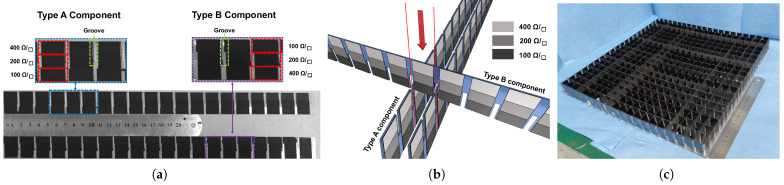
Photographs of the fabricated prototype. (**a**) Graphene films with gradient sheet resistance. (**b**) Schematic of assembly. (**c**) Proposed absorber with 20 × 20 units.

Figure 7a presents a photo of the processed graphene films with gradient sheet resistance on grooved PMMA. In terms of films, our experimental results show that the sheet resistance error using this processing method can be kept below 15%, which is tolerable in the proposed MMA. There are two main reasons for the slight error: uneven coating thickness and deviation caused by multiple manual printing. Both of them are inevitable due to the manual process.

The grooves on PMMA were reserved for assembly as in the schematic in Figure 7b. Two types of components with different grooves were fabricated. For type A, the groove was left on the side of films of 400 Ω/□ . On the contrary, the groove was left on the side of films of 100 Ω/□ for type B. As a result, the prototype was assembled by cross-placing the components and then fixing them to the metallic backboard. Figure 7c shows the proposed absorber assembled with 42 pieces of components in Figure 7a.

### 3.2. Measurement Results

**Figure 8:**
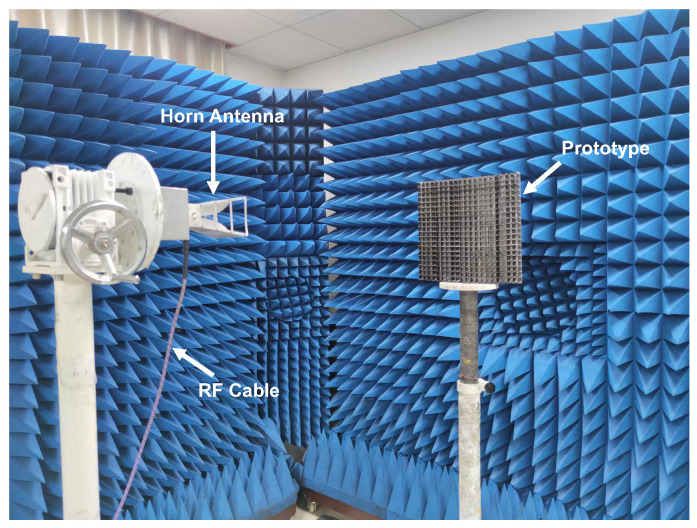
The photograph of the measurement set-up.

The reflection coefficient was measured using the free space method. The measurement set-up is shown in Figure 8. Due to the extremely broad frequency band, the measurement was carried out with two horn antennas of 2–18 GHz and 18–40 GHz, respectively. According to the far field conditions, the electromagnetic energy emitted by the horn antenna will radiate to the sample area in the form of plane waves.

**Figure 9:**
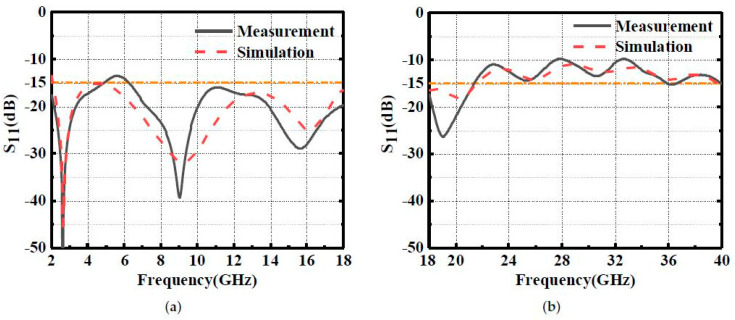
Measurement $S_{11}$ (**a**) 2–18 GHz. (**b**) 18–40 GHz.

The comparison of measurement and simulation results is shown in Figure 9. The results show that the proposed MMA realizes an ultra-broad 10-dB absorption band from 2.0 GHz to 40 GHz. Figure 9a indicates that the $S_{11}$ is still lower than −15 dB at 2 GHz, which agrees with the simulated ones. Further, during 2–21.5 GHz, the absorptivity is larger than 97%, corresponding to the highly-efficient absorption band. The measurement results are in good agreement with the simulated ones, which indicates that the proposed design method of the gradient impedance absorber is effective.

### 3.3. Performance Comparison and Discussion

The performance comparison between the proposed MMA and other reported ultra-broadband absorbers is shown in Table 3. This table records two kinds of absorption bands of those typical absorbers. One is a highly-efficient absorption band with an absorptivity of 97% and the other is a normal absorption band with an absorptivity of 90%. The table indicates that the thickness of 2D absorbers is usually thinner than that of 3D ones, though the bandwidth is not broad enough, and that the absorptivity of the 2D absorbers is lower and they cannot realize a broadband and highly-efficient absorption.

It is well known that a 3D absorber can easily realize ultra-broadband absorption. However, in terms of the highly-efficient absorption with an absorptivity larger than 97%, it is also difficult for 3D absorbers to realize during a broad band. For example, the highly-efficient absorption bands in [2,4,19] are much narrower than their normal absorption bands, while [20] is different. That is because the adopted gradient structure can help to improve the impedance matching at a basic frequency and then raise the absorptivity. However, [20] meets the shortcomings of a narrow operating band, heavy weight and complicated processing.

Compared with those works, the proposed absorber exhibits an ultra-broadband and highly-efficient absorption at the same time due to the stand-up gradient impedance graphene films. The adopted gradient impedance structure maintains the performance at a low frequency efficiently. The stand-up resistive films structure creates the ultra-broad operating band.

## 4. Conclusions

In this paper, we presented an ultra-broadband and highly-efficient metamaterial absorber with stand-up gradient impedance graphene films. The proposed absorber shows the ability of ultra-broadband and highly-efficient absorption. The theory of small reflections was used to help with understanding the better matching brought by gradient impedance. A field analysis was performed to illustrate the mechanism of ultra-broadband absorption. To verify our design, we fabricated a prototype with 20 × 20 units. In fabrication, conductive graphene ink and screen printing technology were adopted. To save processing cost and time, the sheet resistance of graphene in the gradient impedance structure was set as a multiplicative relationship. Then, we could print films with different sheet resistances by changing the print times. The measured results are in good agreement with simulated ones, which indicates that the design method of gradient impedance is effective. The proposed absorber realizes a highly-efficient absorption with an absorptivity larger than 97% during the most commonly used radar band (2–22 GHz), which makes it of great practical value and has application prospects in the field of stealth technologies.

## Figures and Tables

**Table 1:** Parameters of the proposed absorber.

**Parameters**	*w*	*g*	*t*	*p*	*h*	$h_{1}$	$h_{2}$	$h_{3}$	$R_{s}$ ^1^
**Value (mm)**	15.8	1.2	1	17	27	9	9	9	100

^1^ The unit of this parameter is Ω/□.

**Table 2:** The required materials to prepare graphene conducive ink for printing films with a sheet resistance of 400 Ω/□.

Component	Material	Mass (g)
Conductive Filler	GEAS ^1^	46.13
	carbon black particles	2.11
Solvent	deionized water	5.25
	propylene glycol	5.12
Additive agent	CMC ^2^	0.13
	9300 ^3^	0.29
	4600 ^4^	0.26
	XR500 ^5^	0.28
Binder	AR ^6^	4.05

^1^ Graphene electric aqueous slurry. ^2^ Sodium carboxymethyl cellulose CMC-2200 (thickening agent). ^3^ Sodium polyacrylate ACUMER 9300 (dispersant agent). ^4^ Silok 4600 (defoaming agent). ^5^ Epoxy silane (cross-linking agent). ^6^ Acrylic resin (binding agent).

**Table 3:** Performance comparison table between the proposed MMA and other reported ultra-broadband/highly-efficient absorbers.

Refs	Highly-Efficient Absorption Band ^1^ (FBW ^2^)	Normal Absorption Band ^3^ (FBW)	Thickness ($\lambda_{L}$ ^4^)	Structure Type	Features
[2]	4.25–9.88 (79.7%)	3.78–10.47 (93.9%)	0.17	3D, Stand-up patterned ITO	Transparent & Highly-efficient absorption
[3]	8.1–32.2 (119.7%)	1.46–40 (186%)	0.67	3D, Stand-up resistive films	Broadband
[4]	4.2–7.6 (57.6%) & 12.6–30.7 (83.6%)	3.5–31.8 (160%)	0.21	3D, Stand-up Lattice	Transparent & Broadband
[14]	18.1–20.8 & 25.5	3.9–26.2 (148.2%)	0.66	3D, Stand-up resistive films	Broadband & lightweight
[19]	10.3–40 (118%)	2.5–40 (176.5%)	0.65	3D, Stand-up resistive strips	Broadband & lightweight
[20]	8.2–17.2 (70.8%)	8–18 (77%)	0.21	3D, Pyramidal structure	Gradient structure
[21]	N.A. ^5^	6.9–17.4 (86%)	N.A.	3D, Stand-up SSPP ^6^ structure	Wide angular stable
[22]	7.5–20 (90.9%)	6–22 (114%)	0.06	2D	Low profile
[23]	9.4–13.6 (36.5%)	4–14.3 (112.6%)	0.23	2D	/
[24]	N.A.	5.3–18 (108%)	N.A.	2D	/
[25]	N.A.	9.3–49 (136%)	N.A.	2D	Water-based
[26]	1.15	1.1–14.2 (125%)	0.092	2D, multi-layer patterned films	Low profile
Ours	2.0–21.7 (165%) & 39.6–57 (36%)	1.8–66.7 (190%)	0.18	3D, Stand-up gradient films	Broadband & Highly-efficient absorption

^1^ Absorptivity in this band is larger than 97%, $S_{11}$ is lower than −15 dB. The unit is GHz. ^2^ FBW: fractional bandwidth. ^3^ Absorptivity in this band is larger than 90%, $S_{11}$ is lower than −10 dB. The unit is GHz. ^4^ $\lambda_{L}$ is the free-space wavelength at the lowest operating frequency with the absorptivity of 97%. ^5^ Not applicable. This means the absorptivity cannot exceed 97%. ^6^ SSPP: spoof surface plasmon polariton.

## Data Availability

Not applicable.
